# Dysregulation of G protein subunits in autism: decreased GNAO1 and elevated GNAI1 levels in ASD

**DOI:** 10.3389/fpsyt.2025.1587727

**Published:** 2025-08-12

**Authors:** Hatice Topal, Nilfer Sahin, Cilem Ozdemir, Özlem Nehir Yazici, Gulsum Demirkan Baskaya, Yasar Topal, Tuba Edgunlu

**Affiliations:** ^1^ Department of Pediatrics, Faculty of Medicine, Muğla Sıtkı Koçman University, Mugla, Türkiye; ^2^ Department of Child and Adolescent Psychiatry, Faculty of Medicine, Muğla Sıtkı Koçman University, Mugla, Türkiye; ^3^ Department of Bioinformatics, Graduate School of Natural and Applied Sciences, Muğla Sıtkı Koçman University, Muğla, Türkiye; ^4^ Department of Child and Adolescent Psychiatry, Muğla Training and Research Hospital, Mugla, Türkiye; ^5^ Department of Medical Biology, Faculty of Medicine, Muğla Sıtkı Koçman University, Muğla, Türkiye

**Keywords:** autism spectrum disorder, G protein subunits, GNAO1, GNB1, Gnai1

## Abstract

**Objective:**

Autism spectrum disorder (ASD) is a neurodevelopmental condition characterized by social interaction deficits and repetitive behaviors. This study explores the potential role of G protein subunits GNAO1, GNB1, and GNAI1 in the underlying mechanisms by comparing their serum levels in individuals with ASD and non-autistic participants.

**Methods:**

We enrolled 42 children (aged 3–7 years) diagnosed with ASD and 42 age- and sex-matched non-autistic participants. Serum levels of GNAO1, GNB1, and GNAI1 were quantified using ELISA. Additionally, in silico analysis was conducted to investigate protein interaction networks and functional enrichment.

**Results:**

Serum GNAO1 levels were significantly decreased (*p*=0.049), while GNAI1 levels were significantly increased (*p*=0.046) in the ASD group compared to controls. No significant difference was observed in GNB1 levels (*p*=0.141). In silico analysis implicated these proteins in GABAergic and dopamine signaling pathways, both of which are critically involved in neurobiological basis of ASD.

**Conclusions:**

Our findings suggest that dysregulation of G protein signaling pathways, characterized by reduced GNAO1 and increased GNAI1 levels, may contribute to underlying mechanisms of ASD. Further research is warranted to clarify the mechanistic roles of these subunits in ASD and their potential as biomarkers or therapeutic targets.

## 1 Introduction

Autism spectrum disorder (ASD) is a complex neurodevelopmental condition characterized by repetitive, restricted, and inflexible behaviors, as well as deficits in social communication and interaction. While ASD is typically diagnosed in early childhood, the severity and presentation of symptoms can vary significantly among individuals and may evolve across the lifespan (1, 2). The onset of symptoms before the age of three suggests that neuroanatomical and neurochemical alterations during early central nervous system (CNS) development play a critical role in the mechanisms of autism. Neurotransmitters and neuropeptides are essential for normal brain development, regulating key processes such as memory formation, behavior modulation, and motor activity (3). Dysfunctions in these systems can disrupt critical neurodevelopmental processes, including neuronal cell differentiation, migration, apoptosis, and synaptogenesis, which are thought to underlie the neurobiological basis of ASD (4, 5).

G-protein coupled receptors (GPCRs) are transmembrane proteins that mediate cellular responses to neurotransmitters and hormones by activating heterotrimeric G proteins composed of α, β, and γ subunits (6, 7). Among the α-subunits, Gαo (encoded by *GNAO1*) is highly expressed in the nervous system and regulates cAMP synthesis through pathways such as dopamine D2 and GABA-B receptors (8, 9). *GNB1*, encoding the Gβ1 subunit, influences neurotransmitter release by modulating presynaptic calcium and potassium channels (8). *GNAI1*, encoding Gαi1, inhibits adenylate cyclase activity in response to beta-adrenergic signals, thereby reducing cAMP levels (10).

It has been predicted that the active roles of GNAO1, GNB1, and GNAI1 proteins in neuronal function and neurotransmitter release may affect the neurobiological mechanisms underlying ASD (11, 12). Although *GNAO1* gene variants are generally associated with severe epilepsy and motor disorders, the fact that they reduce Gβγ release by disrupting Gαo-mediated dopamine signaling suggests that they may be associated not only with severe neurodevelopmental conditions but also with milder spectrum disorders such as ASD (13). GNAI1-associated neurodevelopmental disorder (GNAI1-NDD) is characterized by developmental delay, intellectual disability, hypotonia, epilepsy, and neurobehavioral symptoms, especially those specific to ASD (stereotyped movements, sensory sensitivity, anxiety, hyperactivity, inattention). ASD or similar neurobehavioral findings have been observed in 30% of reported cases (14). Additionally, *GNAI1* variants are associated with developmental delay, intellectual disability, motor and language delay, hypotonia, and epilepsy. Approximately one-third of individuals carrying the relevant variants are diagnosed with autism (15). Pathogenic variants in *GNB1* have been associated with neurodevelopmental disorders involving developmental delay, intellectual disability, and behavioral symptoms, including autism spectrum disorder. A recently identified mutation predicted to disrupt *GNB1* function further supports its role in synaptic signaling pathways relevant to ASD (16).

Therefore, in this study, we aimed to investigate the potential roles of GNAO1, GNB1, and GNAI1 proteins in the neurobiological mechanisms underlying ASD by comparing their serum levels (as shown in https://www.proteinatlas.org/) between individuals with autism spectrum disorder (ASD) and a non-autistic participants.

## 2 Methods

### 2.1 Study population

Our study included 42 pediatric participants who applied to Muğla Sıtkı Koçman University Faculty of Medicine, Department of Child Health and Diseases and were diagnosed with ASD according to DSM-5 diagnostic criteria. The control group included 42 non-autistic participants without chronic physical or psychiatric diseases. Participants who received medical treatment had any chronic disease or had a psychiatric diagnosis other than a mental disorder were not included in the study. The gender, age, maternal and paternal age of the participants were recorded. The study protocol received approval from the Muğla Sıtkı Koçman University Faculty of Medicine Medical Ethics Committee (decision number 16/I). Informed consent was obtained from the guardians of all child participants prior to the commencement of the study.

### 2.2 Enzyme-linked immunosorbent assay quantification

Blood samples were collected into biochemistry tubes. After 5 ml of blood was collected from each participant for routine biochemical parameters, the blood obtained was centrifuge spinning at 2000 RPM, and the supernatant (serum) was transferred to clean tubes. The obtained serum samples were stored at -80°C. GNAO1 (Cat. No: SL3975HU), GNB1 (Cat. No: SL3993HU) and GNAI1 (Cat. No: SL3994HU) sandwich ELISA kits from SunLong Biotech Co.,LTD were used according to the manufacturer’s instructions (17). Briefly, 50 µL of standard or serum sample was added to each well and incubated for 30 minutes at 37°C with the capture antibody. After washing the wells five times with wash buffer, 50 µL of streptavidin-HRP conjugate was added and incubated again for 30 minutes at 37°C. Subsequently, wells were washed five times, and 50 µL of TMB substrate solution was added and incubated for 15 minutes in the dark at room temperature. Finally, 50 µL of stop solution was added to each well, and the absorbance was immediately measured at 450 nm using a microplate reader. Results were expressed in pg/mL for all proteins.

### 2.3 In silico analysis

We performed in silico analyses to support our experimental findings and hypothesis. We used default parameters when performing the analyses. We determined the possible interaction networks of GNAO1, GNB1 and GNAI1 proteins with STRING version 12.0 (https://string-db.org/) at a medium confidence level. Functional enrichment of pathways associated with the identified networks is also reported by STRING.

### 2.4 Statistical analysis

Statistical analyses were performed using SPSS 22 for Windows (SPSS, Chicago, Illinois, USA). The Shapiro-Wilk test was applied to assess the normality of data distribution for continuous variables. For variables with normal distribution, comparisons between groups were performed using the Independent Sample t-test, while for variables not normally distributed, the Mann-Whitney U test was used. Categorical variables were analyzed using the Chi-square test. Continuous variables are presented as median and interquartile range (IQR) for non-normally distributed data, and mean ± standard deviation (SD) for normally distributed data. Correlation between age and serum protein levels was assessed using the Spearman correlation test. A p-value of less than 0.05 was considered statistically significant.

## 3 Results

### 3.1 Demographic data

There was no statistically significant difference in demographic data between the ASD and control groups (p>0.05) (**Table 1**).

**Table 1:** Demographics data of participants.

Clinical characteristics	Mean ± SD		p value
	ASD (n=42)	Control (n=42)	
Age	3.90 ± 1.54	4.46 ± 2.70	0.269*
Gender, n(%)			
Male	33 (78.57)	27 (64.29)	0.147**
Female	9 (21.42)	15 (35.71)	
Maternal Age	33.69 ± 6.07	33.16 ± 6.02	0.719*
Paternal Age	37.45 ± 6.16	36.06 ± 5.80	0.338*

*Independent sample t-test, **Pearson chi-square, SD, Standard deviation.

### 3.2 Protein levels of GNAO1, GNB1 and GNAI1

**Figure 1:**
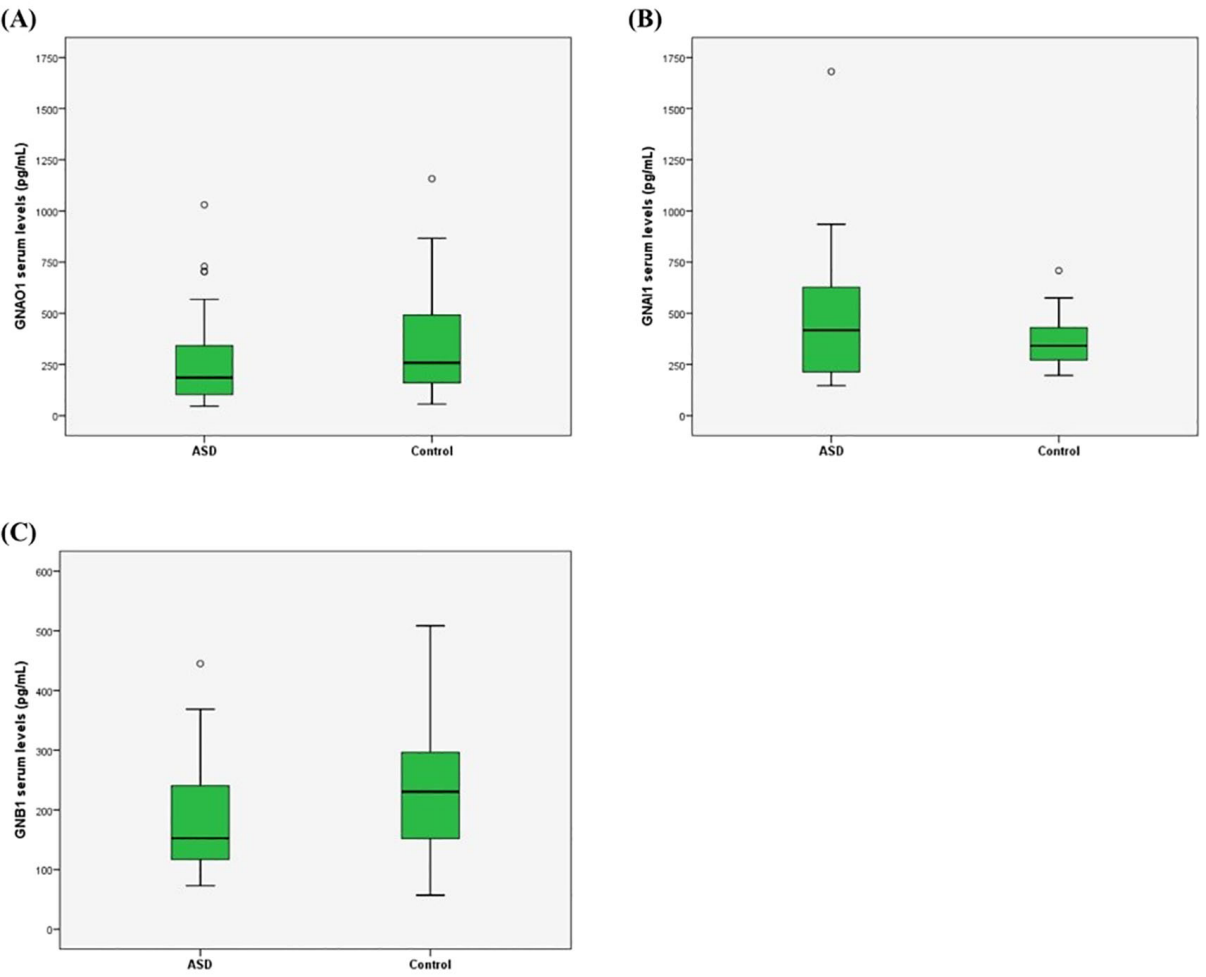
GNAO1 **(A)**, GNAI1 **(B)**, and GNB1 **(C)** serum levels. The levels of GNAO1 and GNAI1 expression in serum level of ASD cases and controls were significantly different (p=0.049, p=0.046 and p=0.141, respectively).

We determined the protein levels of GNAO1, GNB1, and GNAI1 in the ASD and control groups. While the levels of GNAO1 and GNAI1 were different between the ASD and control groups (p=0.049 and p=0.046, respectively) the levels of GNB1 were no different between the two groups (p=0.141) (**Table 2**, **Figure 1**). There were no significant differences in the serum protein levels between genders (p=0.673 for GNAO1, p=0.080 for GNAI1 and p=0.935 for GNB1). No significant correlation was found between age and serum levels of GNAO1, GNB1, or GNAI1 (p > 0.05 for all comparisons).

**Table 2:** GNAO1, GNB1 and GNAI1 protein levels.

	ASD (n=42)	Control (n=42)	
Protein	Median(IQR)	Median(IQR)	p* value
GNAO1	185.61 (248.07)	260.81 (384.95)	**0.049**
GNAI1	452.49 (456.08)	346.91 (158.25)	**0.046**
GNB1	158.54 (127.93)	229.23 (161.79)	0.141

*Mann-Whitney U test, IQR, Interquartile range.

Bold values are statistically significant.

### 3.3 In silico analysis

Using interactions between GNAO1, GNB1, and GNAI1 we performed functional enrichment analysis for four levels of putative STRING networks (**Table 3**). Only ASD-related results from the KEGG and Gene Ontology databases were included in the table. The first pathway identified in the gene ontology of the first level was the Adenylate cyclase-modulating G protein-coupled receptor signaling pathway. Other network levels consist of a total of 11 genes, and the gene ontology results included Negative regulation of the dopamine receptor signaling pathway, Adenylate cyclase-activating dopamine receptor signaling pathway, and Negative regulation of dopamine secretion pathways, respectively. The pathway primarily identified in KEGG at all network levels was the GABAergic synapse signaling pathway. According to the analysis results, GNAO1, GNB1 and GNAI1 networks appear to play functional roles in ASD-related cellular processes.

**Table 3:** Functional enrichments of networks based on GNAO1, GNB1 and GNAI1 interactions.

	Network	Proteins	Network stats	Database	Description	Strength^1^	FDR^2^
1	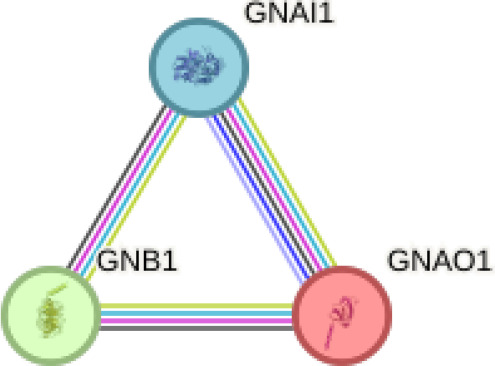	GNAO1 GNB1 GNAI1	number of nodes: 3 number of edges: 3 average node degree: 2 avg. local clustering coefficient: 1 expected number of edges: 0 PPI enrichment p-value: 0.0029	Gene Ontology	1# GO:0007188 Adenylate cyclase-modulating G protein-coupled receptor signalling	1.93	0.0263
				KEGG Pathways	1# hsa:04727 GABAergic synapse	2.19	0.0194
2	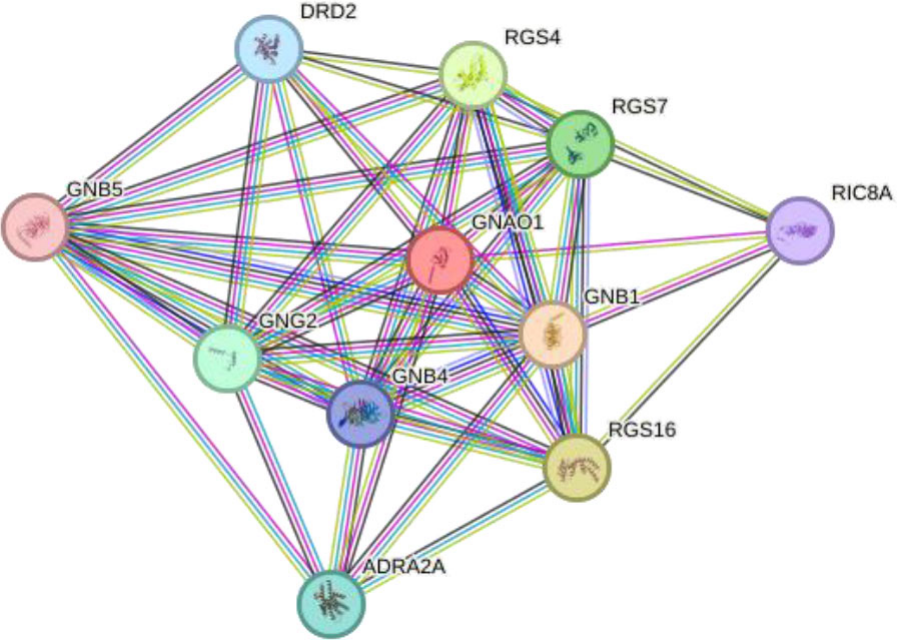	GNAO1 GNB1 GNB4 GNG2 GNB5 ADRA2A RGS16 RGS4 RGS7 DRD2 RIC8A	number of nodes: 11 number of edges: 46 average node degree: 8.36 avg. local clustering coefficient: 0.893 expected number of edges:12 PPI enrichment p-value: 1.15e-13	Gene Ontology	1# GO:0060160 Negative regulation of dopamine receptor signaling pathway	3.08	0.0014
				KEGG Pathways	1# hsa04727- GABAergic synapse	1.93	1.40e-05
3	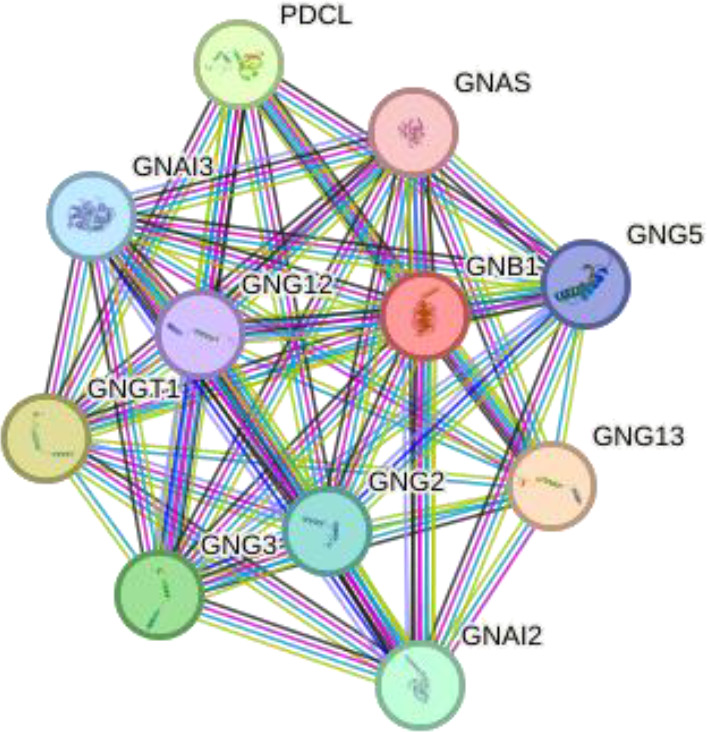	GNB1 GNG5 GNG12 GNG13 GNG2 GNG3 GNGT1 GNAI2 GNAS GNAI3 PDCL	number of nodes: 11 number of edges: 52 average node degree: 9.45 avg. local clustering coefficient: 0.958 expected number of edges: 14 PPI enrichment p-value: 1.42e-14	Gene Ontology	1# GO:0007191 Adenylate cyclase-activating dopamine receptor signaling pathway	2.73	0.00019
				KEGG Pathways	3# hsa04727- GABAergic synapse	2.28	1.97e-18
4	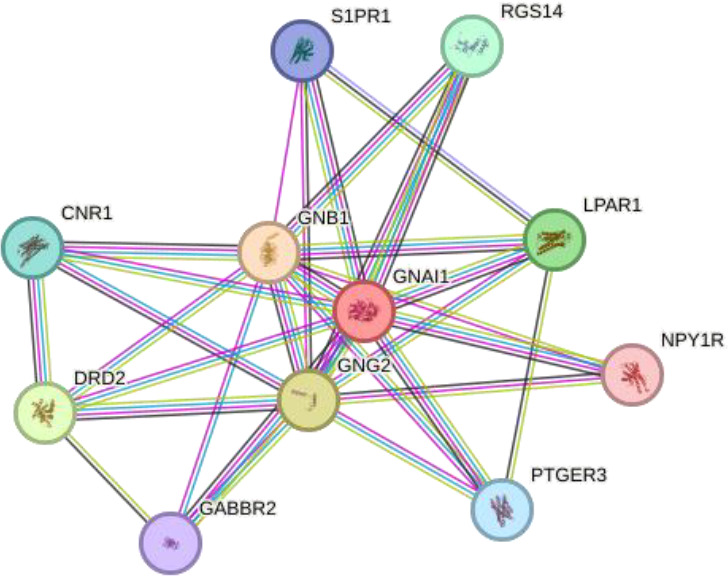	GNAI1 LPAR1 GNG2 GABBR2 GNB1 DRD2 CNR1 S1PR1 RGS14 NPY1R PTGER3	number of nodes: 11 number of edges: 52 average node degree: 9.45 avg. local clustering coefficient: 0.958 expected number of edges: 14 PPI enrichment p-value: 1.42e-14	Gene Ontology	1# GO:0033602 Negative regulation of dopamine secretion	2.86	0.0067
				KEGG Pathways	2# hsa04727 GABAergic synapse	1.93	1.05e-05
	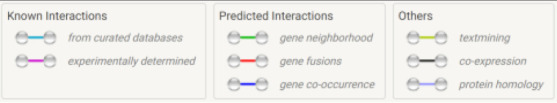		^1^Strength: Log10(observed/expected). This measure describes how large the enrichment effect is. It’s the ratio between i) the number of proteins in your network that are annotated with a term and ii) the number of proteins that we expect to be annotated with this term in a random network of the same size. ^2^False Discovery Rate: This measure describes how significant the enrichment is. Shown are p-values corrected for multiple testing within each category using the Benjamini–Hochberg procedure.				

## 4 Discussion

ASD, a neurodevelopmental disorder, is characterized by social communication and interaction deficits and repetitive and stereotyped behaviors. Considering the complexity of underlying mechanisms and its increasing prevalence, there is a need to identify biomarkers for treatment or diagnosis. G protein subunits play critical roles in neuronal processes (synaptogenesis, neurotransmitter release, neuronal migration, etc.) necessary for brain development and function in the nervous system (18). This study investigated serum G protein subunits GNAO1, GNB1 and GNAI1 levels among individuals with ASD and non-autistic participants. According to our results, a significant decrease in GNAO1 values (p = 0.049) and a significant increase in GNAI1 values (p = 0.046) were observed in ASD participants compared to controls but GNB1 did not differ (p=0.141). In addition, according to in silico analysis, GABAergic and dopamine signaling pathways were among the prominent results in protein interaction networks.

The observed decrease in GNAO1 and increase in GNAI1 levels in individuals with ASD are potentially indicative of an imbalance in excitatory and inhibitory signaling. GNAO1 is associated with excitatory G protein-coupled receptor (GPCR) signaling, while GNAI1 is associated with inhibitory signaling pathways. In ASD, there is a well-documented imbalance between excitatory and inhibitory neurotransmission, which is thought to contribute to many of the disorder’s clinical features (19). The reduction in GNAO1 levels may impair excitatory signaling, particularly in dopaminergic and GABAergic pathways, which are crucial for social behavior, communication, and motor control. On the other hand, the increase in GNAI1 could enhance inhibitory signaling, further exacerbating the excitatory-inhibitory imbalance. While our study found no significant differences in GNB1 levels between ASD participants and non-autistic participants, the potential role of GNB1 in neurodevelopmental disorders should not be overlooked. Mutations in *GNB1* have been associated with developmental delay, hypotonia, and various behavioral disorders (16, 20).

Considering the interaction networks, the prominent signaling pathway at every level was the GABAergic signaling pathway. According to GO analyses, the processes that regulate dopamine secretion attracted attention. The GNAO1, GNB1 and GNAI1 proteins included in the research may be effective in the underlying mechanisms of ASD. Studies have revealed the effect of dopamine release and GABAergic signaling pathways on ASD. Gamma-aminobutyric acid (GABA) plays a role in the neurodevelopment and regulation of neuronal activities in the central nervous system. It shows that GABAergic signaling dysregulation may be responsible for many of the clinical symptoms in autistic individuals (19, 21). Dopamine is a neurotransmitter that plays important roles in motor control, social behavior and cognition. It has been stated that dopaminergic dysfunction in certain areas of the brain can lead to autistic behaviors (4, 22, 23). While our data indicate reduced serum GNAO1 levels in ASD, the potential impact on dopaminergic and GABAergic signaling remains requires further investigation. This may include difficulties in communication and social interaction, repetitive relationships, and limited interests, which can be found among the symptoms of ASD. An increase in GNAI1 levels may lead to other neurocognitive abnormalities that may contribute to ASD as it would activate inhibitory G protein signaling pathways.

To better understand the relevance of the interaction network of GNAO1, GNB1, and GNAI1 in ASD, we explored whether other genes in the same protein–protein interaction network (as identified through STRING) have previously been associated with ASD. Our STRING-based interaction analysis revealed several proteins functionally connected to GNAO1, GNB1, and GNAI1, such as LPAR1, GABBR2, DRD2, DRD1, and others. Previous studies have shown that alterations in these genes may contribute to cognitive impairments, neurotransmitter imbalance, and synaptic dysfunctions commonly observed in ASD. *LPAR1* was associated with cognitive deficits and negative behavioral deficits in mouse models with ASD (24). It has been suggested that decreased *GABBR2* expression in the cerebellum and the variants in the *GABBR2* gene may be effective in the neurobiological basis of autism (25–27). *DRD2* and *PPP1R1B* genes have been identified as risk factors for ASD in families with only affected males (28). It has been found that polymorphisms in DRD1 and DRD2 receptors may contribute to the risk of ASD (29). An association between *DRD2* polymorphisms and prolactin levels has been demonstrated in ASD participants treated with risperidone (30). Studies highlight the importance of the dopaminergic system in neurobiological basis of ASD. *RGS14* gene expression did not change in ASD participants (31). It has been reported that chromosome 4q deletion contributed to hemizygosity of *NPY1R*, one of the neuropeptide receptors, in a child with ASD (32). The *ADRA2A* variant exhibited statistically significant differences between autistic individuals who responded and did not respond to methylphenidate treatment, and the variant in DRD2 was associated with tolerability (33). To our knowledge, there are no studies explaining the contribution of *CNR1, S1PR1, PTGER3, RIC8A, RGS7, RGS4, GNB4, GNB5, RGS16, GNG12, GNG2, GNG3, GNGT1, GNAI2, GNAI3, GNAS, PDCL* genes to underlying mechanisms of ASD. The genes in the interaction network may be new target genes for ASD and further studies are needed with these genes. Our findings showing altered serum levels of GNAO1, GNB1, and GNAI1 may therefore reflect a broader dysregulation within this network, warranting further investigation into these understudied genes.

Although our study identified altered levels of GNAO1 and GNAI1, it is important to note that current research directly linking these G protein subunits to ASD is still limited and inconclusive. This study is limited by its reliance on serum protein levels, which may not fully reflect the expression or activity of GNAO1, GNB1 and GNAI1 in the central nervous system (CNS). The absence of data from brain tissues, cerebrospinal fluid (CSF), or *in vivo* assays restricts our ability to directly link these findings to neurodevelopmental processes in ASD. Future studies incorporating CNS-specific samples are needed to validate these results.

## 5 Conclusion

This study highlights altered serum levels of GNAO1 and GNAI1 in individuals with ASD, suggesting their potential role in the neurobiological basis of ASD, particularly in excitatory-inhibitory imbalances affecting dopamine and GABA signaling. However, the use of serum limits direct CNS insights, necessitating further research with brain-specific tissues and *in vivo* analyses to confirm these findings. While GNB1 showed no significant changes, its potential role in neurodevelopmental disorders warrants additional investigation.

## Data Availability

The raw data supporting the conclusions of this article will be made available by the authors, without undue reservation.
